# Effect on Antimicrobial Resistance of a Policy Restricting Over-the-Counter Antimicrobial Sales in a Large Metropolitan Area, São Paulo, Brazil

**DOI:** 10.3201/eid2801.201928

**Published:** 2022-01

**Authors:** Maria L. Moura, Icaro Boszczowski, Manuela Blaque, Rafael M. Mussarelli, Victor Fossaluza, Ligia C. Pierrotti, Gustavo Campana, Maria C. Brandileone, Rosemeire Zanella, Samanta C.G. Almeida, Anna S. Levin

**Affiliations:** Universidade de São Paulo Hospital das Clínicas, São Paulo, Brazil (M.L. Moura, I. Boszczowski);; Universidade de São Paulo Instituto de Matemática e Estatística, São Paulo (M. Blaque, R.M. Mussarelli, V. Fossaluza);; Diagnósticos da América Laboratory, São Paulo (L.C. Pierrotti, G. Campana);; National Laboratory for Meningitis and Pneumococcal Infections, São Paulo (M.C. Brandileone, R. Zanella, S.C.G. Almeida);; Universidade de São Paulo Faculdade de Medicina, São Paulo (A.S. Levin)

**Keywords:** antimicrobial resistance, antimicrobial drugs, anti-infective agents, nonprescription drugs, over-the-counter drugs, bacteria, public policy, public health, government regulation, Brazil

## Abstract

Although restricting over-the-counter (OTC) antimicrobial drug sales is recommended globally, no data track its effect on antimicrobial resistance (AMR) in bacteria. We evaluated the effect of a national policy restricting OTC antimicrobial sales, put in place in November 2010, on AMR in a metropolitan region of São Paulo, Brazil. We reviewed associations between antimicrobial sales from private pharmacies and AMR in 404,558 *Escherichia coli* and 5,797 *Streptococcus pneumoniae* isolates using a dynamic regression model based on a Bayesian approach. After policy implementation, a substantial drop in AMR in both bacterial species followed decreased amoxicillin and trimethoprim/sulfamethoxazole sales. Conversely, increased ciprofloxacin sales were associated with increased ciprofloxacin resistance, and extended spectrum β-lactamases–positive *E. coli* isolates and azithromycin sales increases after 2013 were associated with increased erythromycin resistance in *S. pneumoniae* isolates. These findings suggest that restricting OTC antimicrobial sales may influence patterns of AMR, but multifaceted approaches are needed to avoid unintended consequences.

The spread of antimicrobial resistance (AMR) is a global concern that requires multilevel efforts because of its serious public health consequences (*1*). Misuse of and overexposure to antimicrobial drugs are considered major factors that accelerate the emergence of multidrug-resistant organisms. Inappropriate prescription and self-medication contribute to overuse of antimicrobial drugs and might promote emergence of antimicrobial drug–resistant bacteria. In response, the World Health Organization recommends regulating use of antimicrobial drugs by restricting over-the-counter (OTC) sales, an approach that has been implemented in many low- and middle-income countries in the past decade (*2*,*3*). Although this is a global recommendation, data are scarce on the effect of restricting antimicrobial sales on AMR.

In November 2010, the National Health Surveillance Agency of Brazil (Agência Nacional de Vigilância Sanitária [ANVISA], https://www.gov.br/anvisa/pt-br) implemented a restriction policy requiring a medical prescription to purchase antimicrobials (*4*); OTC sales had been common before the policy took effect. The restriction policy reduced antimicrobial sales in private pharmacies, but no data have shown its effect on AMR (*5*). We evaluated the effect of this policy during 2008–2016 on AMR in 2 bacteria that frequently cause community-acquired infections before and after the restriction policy was initiated. 

## Methods

### Study Area

The São Paulo, Brazil, metropolitan region is the largest metropolitan area in Latin America and the largest industrial and commercial hub in Brazil. It includes the city of São Paulo and 38 other municipalities, comprising a geographic area of 7,946 km^2^ and 21.6 million inhabitants, a population greater than that of many countries in the world (*6*) and that constitutes ≈50% the population of the state of São Paulo. The human development index in the São Paulo metropolitan region was 0.78 in 2013, which is higher than that of the other states in Brazil. São Paulo state has 2.81 physicians/1,000 inhabitants, Brazil’s second highest rate, and 38% of its population has private health insurance, compared with 23% nationally (*7*).

### Antimicrobial Drug Sales

For this study, we analyzed data on monthly antimicrobial drug sales from private pharmacies in the São Paulo metropolitan region from 2008 through 2016. Data on antimicrobial sales were obtained from audits performed by IQVIA Brazil (https://www.iqvia.com), an international company that performs pharmaceutical industry marketing research. From 2008 through 2012, data were purchased directly from IQVIA Brazil, funded by the São Paulo Research Foundation (Fundação de Amparo à Pesquisa do Estado de São Paulo, https://fapesp.br). After March 2013, the data were provided by Pfizer Brazil (https://www.pfizer.com), through a formal agreement with the University of São Paulo. Pfizer had previously purchased the information on sales from private pharmacies from IQVIA Brazil for marketing purposes. We evaluated data on 6 oral antimicrobials: amoxicillin, azithromycin, cephalexin, ciprofloxacin, nitrofurantoin, and trimethoprim/sulfamethoxazole (cotrimoxazole).

In our analyses, we excluded public healthcare services, such as primary care units, outpatient clinics, and hospitals supported by the government, because they already required medical prescriptions for antimicrobial drug sales before the national restriction policy went into effect. A previous study demonstrated that the restriction policy did not affect antimicrobial consumption by patients of those public sector agencies (*5*). Public sector agencies represent 14.5% of healthcare facilities in São Paulo state and are responsible for the healthcare of 61% of the population (*8*). In the São Paulo metropolitan region, antimicrobial consumption by patients in these sectors increased from 2008 to 2012, but this increase did not affect overall antimicrobial sales (Appendix Table). After the restriction policy, all private pharmaceutical businesses were required to report antimicrobial sales to ANVISA using the electronic system of the National System for the Management of Controlled Products (Sistema Nacional de Gerenciamento de Produtos Controlados [SNGPC], https://www.gov.br/anvisa/pt-br/assuntos/fiscalizacao-e-monitoramento/sngpc).

The standard unit for measuring consumption, defined daily doses (DDD) per 1,000 inhabitants per day (DID), used in accordance with the Anatomical Therapeutic Chemical classification system (*9*), is calculated as the quantity of antimicrobials in a given month × 1,000/30 days × DDD for that drug × population. We obtained each year’s population estimate for the São Paulo metropolitan region from the Brazilian Institute of Geography and Statistics website (Instituto Brasileiro de Geografia e Estatística [IBGE], https://www.ibge.gov.br) (*6*).

The study was approved by the Ethics Committee of the University of São Paulo Medical School, São Paulo, Brazil (number 2.608.800). The databases contained no personally identifiable information.

### AMR in Bacteria

To evaluate the effect of the restriction policy on AMR in bacteria, we analyzed the proportion of antimicrobial-resistant bacterial samples from databases of *Escherichia coli* isolates in the São Paulo metropolitan area and *Streptococcus pneumoniae* isolates in São Paulo state. These databases contained information on isolates from patients of all ages in inpatient and outpatient health services.

A clinical laboratory, Diagnostics of America (DASA; https://dasa.com.br), provided *E. coli* isolates through a formal agreement with the University of São Paulo. In São Paulo state, DASA has 288 public and private units, performs 90 million tests per year, and provides medical services for ≈1.8 million people. The database included *E. coli* isolates from urine and blood samples taken by outpatient and inpatient services in the of São Paulo metropolitan area during 2008–2016. It was not possible to define the proportion of the isolates from hospitalized patients.

The database of *S. pneumoniae* isolates was provided by the Bacteriology Center of Adolfo Lutz Institute (Instituto Adolfo Lutz [IAL], http://www.ial.sp.gov.br), located in the city of São Paulo. IAL, the National reference laboratory for *S. pneumoniae* in Brazil, receives isolates from a network of public health reference laboratories and private hospitals all over the country. IAL performs serotyping, antimicrobial susceptibility testing, and molecular typing of all isolates obtained from invasive pneumococcal disease. Also, IAL is part of SIREVA (Sistema de Redes de Vigilancia de los Agentes Responsables de Neumonias y Meningitis Bacterianas [Regional System for Vaccines]), a surveillance system for invasive bacterial diseases initiated in the Americas in 1993 by the Pan American Health Organization (PAHO; https://www.paho.org) (*10*). This database contains susceptibility profiles of isolates, mainly from blood, cerebrospinal fluid, and respiratory samples for all age groups in São Paulo state. During analysis of the impact of the restriction policy on *S. pneumoniae*, we accounted for national introduction of a free-of-charge 10-valent pneumococcal vaccine (PCV10) in March 2010.

### Susceptibility Testing

For *E. coli*, we performed susceptibility testing for cephalothin, amoxicillin, ciprofloxacin, ceftriaxone, nitrofurantoin, and trimethoprim/sulfamethoxazole using a VITEK 2 automated system (bioMérieux, https://www.biomerieux-diagnostics.com) according to Clinical and Laboratory Standards Institute (CLSI; https://clsi.org) criteria (*11*). For the analysis, we assumed nonsusceptible strains to be resistant. Because CLSI changed breakpoints for ceftriaxone in 2010, we considered the phenotypic detection of extended spectrum β-lactamases (ESBL) during the study period to investigate the association between third-generation cephalosporin resistance and antimicrobial sales (*12*). We considered *E. coli* isolates susceptible to amoxicillin if MIC ≤8 for ampicillin. For *S. pneumoniae*, we determined susceptibility to erythromycin and trimethoprim/sulfamethoxazole by disk-diffusion (OXOID, http://www.oxoid.com). To determine penicillin and ceftriaxone susceptibility, we screened for oxacillin susceptibility using disk diffusion according to CLSI guidelines (*11*). For isolates found resistant to any drug by disk diffusion, we determined MIC by broth microdilution to confirm susceptibility status.

### Statistical Analysis

We analyzed the association between monthly resistance rates of *E. coli* isolates to amoxicillin, sulfamethoxazole/trimethoprim, ciprofloxacin, and nitrofurantoin and corresponding sales of those same antimicrobial drugs. Because cephalothin resistance correlates with resistance to first-generation cephalosporins, we investigated its association with sales of cephalexin, the first-generation cephalosporin accounting for the most sales, which is available in oral form. We also analyzed the association between the proportion of ESBL-positive *E. coli* isolates and sales of ciprofloxacin and cephalexin. For *S. pneumoniae*, we evaluated the association between penicillin resistance and amoxicillin sales, erythromycin resistance and azithromycin sales, and trimethoprim/sulfamethoxazole resistance and sales.

We used a dynamic regression model based on a Bayesian approach to analyze the effect of the restriction policy on the association between antimicrobial sales and resistance (*13*). In this model, the estimatedβ values represent the association between AMR and sales. A β value >0 indicates a direct association between AMR and sales and a β value <0 indicates an inverse association (Appendix); β value = 0 indicates no association. Using the dynamic regression analysis, we could estimate different β values at different instants of time, which was notable because it had not been determined how long a reduction in antimicrobial sales takes to influence AMR. This method enabled us to evaluate the effect of policy restrictions on AMR even if this effect did not occur immediately after implementation. For each analysis of AMR relative to sales, we plotted the estimated β values and 95% credible intervals (CrIs) in a graph. We considered that there was an association between antimicrobial sales and resistance in a period if the 95% CrIs did not include 0. We performed analysis using R software version 3.5.1 (https://cran.r-project.org/bin/windows/base/old/3.5.1).

## Results

During the study period, sales of the oral antimicrobial drugs we studied in the São Paulo metropolitan region decreased from 7.86 to 7.65 DID, and we observed a pronounced drop in sales after the 2010 implementation of the restriction policy. Amoxicillin and trimethoprim/sulfamethoxazole accounted for the most sales, ≈3 times the sales of other drugs (Table).

**Table:** Sales of oral antimicrobial drugs in the of São Paulo metropolitan area, Brazil, 2008–2016*

Antimicrobial drug	Antimicrobial sales, DID				
	2008	2010	2012	2014	2016
Amoxicillin	3.22	3.43	2.73	2.37	2.58
Trimethoprim/sulfamethoxazole	2.57	2.87	2.04	2.23	2.19
Ciprofloxacin	0.52	0.68	0.59	0.81	0.88
Azithromycin	0.62	0.66	0.37	0.64	0.86
Cephalexin	0.54	0.70	0.56	0.65	0.63
Nitrofurantoin	0.39	0.43	0.42	0.49	0.51
Total	7.86	8.77	6.71	7.19	7.65

*DID, defined daily dose per 1,000 inhabitants per day.

### AMR of *Escherichia coli*

**Figure 1:**
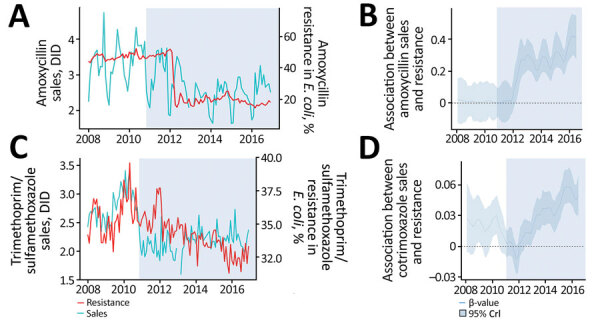
Descriptive analysis of amoxicillin and trimethoprim/sulfamethoxazole sales and *Escherichia coli* resistance in the São Paulo metropolitan area, Brazil, before and after a national policy restricting over-the-counter antimicrobial sales began. A, B) Amoxicillin; C, D) sulfamethoxazole/trimethoprim. Panels B and D show distribution of estimated β values obtained from dynamic regression model, representing the association between drug sales and resistance for *E. coli*. Positive estimated β values and 95% CrI >0 indicate a direct association between sales and resistance. Light blue shaded areas represent period after the restriction policy began. CrI, credible interval; DID, defined daily dose/1,000 inhabitant-days.

We analyzed the susceptibility profile of 404,558 *E. coli* isolates during 2008–2016, 99.5% from urine samples, to assess the association between sales of amoxicillin and trimethoprim/sulfamethoxazole and resistance of *E. coli* to these drugs (Figure 1). After the November 2010 initiation of the restriction policy, amoxicillin sales fell, followed in 2012 by a drop in resistance. Positive estimated β values after 2012 (Figure 1, panel B) demonstrated a direct association between sales and resistance and a notable impact of the restriction policy on AMR. A similar pattern was observed for resistance to sulfamethoxazole/trimethoprim. 

**Figure 2:**
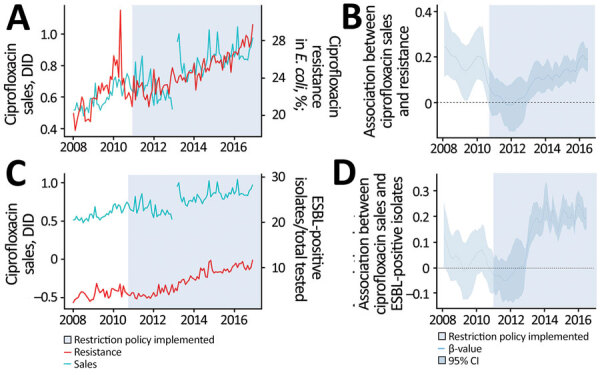
Descriptive analysis of ciprofloxacin sales and *Escherichia coli* resistance in the São Paulo metropolitan area, Brazil, before and after a national policy restricting over-the-counter antimicrobial sales began. A, B) Ciprofloxacin sales and resistance in *E. coli*; C, D) Ciprofloxacin sales and proportion of ESBL-positive isolates. Panels C and D show distribution of estimated β-values obtained from dynamic regression model, representing the association between ciprofloxacin sales and resistance for *E. coli* and proportion of ESBL-positive isolates. A β-value and 95% CrI >0 indicate a direct association between sales and resistance, except for the period between 2011 and 2013. Estimated β values >0 before and after the policy began indicate no influence of the regulation on ciprofloxacin resistance. Light blue shaded areas represent period after the restriction policy began. CrI, credible interval; DID, defined daily dose/1,000 inhabitant-days; ESBL, extended spectrum β-lactamases.

Analysis of the distribution of estimated β values (Figure 2, panel B) suggested an association between ciprofloxacin sales and AMR for *E. coli*, as well as the prevalence of ESBL-positive isolates, but there was no effect from the restriction policy on ciprofloxacin sales; sales continued to increase after the policy was implemented. The increase in ciprofloxacin sales was also associated with an increase in ESBL-positive isolates after the policy restriction took effect. We observed a consistent rise in nitrofurantoin sales throughout the study period, which after the implementation of the restriction policy was significantly associated with resistance (Appendix Figure 1). We observed no significant association between cephalexin sales and cephalothin resistance for *E. coli* or with the proportion of ESBL-positive isolates (Appendix Figures 2, 3).

**Figure 3:**
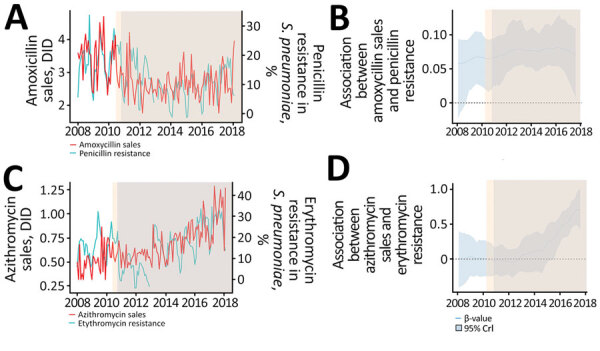
Descriptive analysis of the association between amoxicillin and azithromycin sales and *Streptococcus pneumoniae* resistance to penicillin and erythromycin in the São Paulo metropolitan area, Brazil, before and after a national policy restricting over-the-counter antimicrobial sales began. A, B) Amoxicillin sales and penicillin resistance; C, D) azithromycin sales and erythromycin resistance. Panels B and D show distribution of estimated β-values obtained from dynamic regression model. Estimated β-values and 95% CrI >0 suggest a direct association between sales and resistance before and after the restriction policy began. Penicillin resistance decreased after the restriction policy began (light blue shading areas) and after addition of free-of-charge PCV10 (orange shaded areas) to the national immunization program, and there was a direct association between sales of azithromycin and resistance to erythromycin 1 year after the restriction policy was put in place. CrI, credible interval; DID, defined daily dose/1,000 inhabitant-days; PCV10, 10-valent conjugated pneumococcal vaccine.

### AMR of *Streptococcus pneumoniae*

During the study period, we analyzed 5,797 *S. pneumoniae* isolates: 68.16% from blood, 25.7% from cerebrospinal fluid, 4.5% from respiratory samples, and 1.64% from other sites. Because penicillin sales were much lower than amoxicillin sales, we used amoxicillin sales data to investigate its association with penicillin resistance. Similarly, we used azithromycin sales data to investigate erythromycin resistance in *S. pneumoniae*.

**Figure 4:**
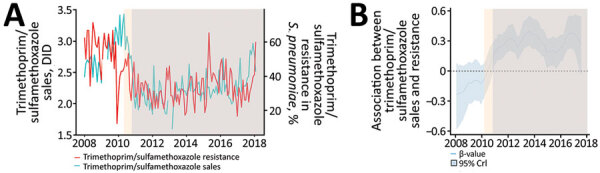
Descriptive analysis of the association between trimethoprim/sulfamethoxazole sales and *Streptococcus pneumoniae* resistance in the São Paulo metropolitan area, Brazil, before and after a national policy restricting over-the-counter antimicrobial sales began. A) Trimethoprim/sulfamethoxazole sales and *S. pneumoniae* resistance; B) distribution of estimated β-values obtained from dynamic regression model. Estimated β values and 95% CrIs >0 indicate a direct association between sales and resistance that starts after the restriction policy was put in place (light blue shaded areas) addition of free-of-charge PCV10 (light orange shaded areas) to the national immunization program and restriction policy in 2010. CrI, credible interval; DID, defined daily dose/1,000 inhabitant-days; PCV10, 10-valent conjugated pneumococcal vaccine.

We found a direct association between antimicrobial sales and resistance in *S. pneumoniae* (Figures 3, 4). During the study period, a substantial decrease in amoxicillin sales was followed by decreased penicillin resistance. Association between amoxicillin sales and penicillin resistance occurred in the periods before and after policy initiation. Azithromycin and trimethoprim/sulfamethoxazole sales showed a direct association with both erythromycin and trimethoprim/sulfamethoxazole resistance, which became more pronounced after the restriction policy began and PCV10 was added to the Brazil National Immunization Program.

## Discussion

Although many studies have correlated antimicrobial consumption and resistance, data are scarce about whether AMR can be reduced by decreasing antimicrobial sales in a community setting (*14*–*16*). Our study suggests that a policy to ban OTC sales of antimicrobial drugs may have influenced a decrease in AMR in a large population.

There are complex mechanisms involved in AMR development; therefore, we felt that it was not possible to predict how long after the change in antimicrobial sales an effect on resistance could be expected in a large population. The advantage of using a dynamic regression model instead of a time-series analysis was the possibility of detecting an association between sales and resistance over any time period after the restriction policy was initiated. Our statistical model enabled us to demonstrate both the association between antimicrobial sales and resistance and the effect of the restriction policy on OTC sales. Most of our data indicated that the effect of the policy on the association of antimicrobial sales and resistance occurred 1 year after its mid-2012 implementation.

We chose to study the main bacteria that caused community-acquired respiratory and urinary tract infections (UTI). For *E. coli,* the main cause of community-acquired UTI, we observed a marked decrease in resistance to amoxicillin and trimethoprim/sulfamethoxazole associated with a decrease in sales of these drugs. Our data suggest that the association between drug sales and resistance might have been affected by the restriction policy. Although the policy appeared to have had no effect on sales of ciprofloxacin and other quinolones, the association between sales and resistance remained; an increase in ciprofloxacin sales was associated with an increase in the proportion of ESBL-positive isolates. These data are very alarming and might reveal an unintended consequence of the restriction policy, shifting antimicrobial consumption towards antimicrobials with higher resistance potential, the opposite of what the World Health Organization considers appropriate antimicrobial consumption according to its AWaRe (access, watch, reserve) classification of antimicrobials (*17*). Observational studies have documented a higher prevalence of ESBL in ciprofloxacin-resistant *Enterobacteriaceae*, and recent data suggest that this association may be plasmid-mediated (*18*,*19*). Because quinolones provide convenient dosing and adequate spectrum to treat common community-acquired infections, physician preference for the drugs and lack of awareness about undesirable consequences may explain the increase in quinolone sales despite the effect of the restriction policy. This finding highlights the need for multifaceted approaches to improve considered use of antimicrobial drugs and decrease AMR. 

A previous study using a retrospective observational design evaluated the effect of the restriction policy on *E. coli* resistance rates from urine samples collected at a teaching hospital in another large metropolitan region of São Paulo state (*20*). The authors analyzed yearly resistance rates from 2009 to 2015 and found no differences after the policy, despite the decrease in antimicrobial consumption immediately after it began. Of note, that study had important methodological differences from our study. First, the outcome was assessed strictly within a tertiary-care hospital, which may have biased the occurrence of resistance. Furthermore, there might be differences between the 2 metropolitan areas in terms of socioeconomic determinants and access to and quality of healthcare services. Adjusting results based on these differences would be interesting.

We observed a direct association between antimicrobial sales for *S. pneumoniae* and resistance for all drugs analyzed. For amoxicillin, we observed that the association between sales and resistance occurred even before the policy took effect. Also, it is important to take into account the introduction of PCV10 as part of the Brazil National Immunization Program in March 2010, which was associated with a 95.5% decrease in colonization by vaccine serotypes in persons <24 years of age in the São Paulo metropolitan region (*21*). The reduction in pneumococcal infections among children after PCV10 was introduced might be associated with decreased consumption of amoxicillin and, therefore, decreased amoxicillin resistance, similar to experiences in other countries (*22*,*23*). On the other hand, after the PCV10 introduction, a higher prevalence was observed of serotype 19 pneumococcal infections, an infection previously associated with higher resistance to penicillin and ceftriaxone (*21*,*24*). Thus, the interactions among pneumococcal vaccination, reduction of antimicrobial use, and antimicrobial resistance require further investigation.

Although the restriction policy was associated with a decrease in trimethoprim/sulfamethoxazole and penicillin resistance, we observed an increase in azithromycin sales beginning in 2013. Similar to quinolone use for UTIs, macrolides are commonly prescribed for upper respiratory tract infections and are one of the first choices for treating community-acquired pneumonia in Brazil (*25*). An evaluation of physicians’ prescription drug choices and awareness of AMR in Brazil is important to elucidate this hypothesis. Azithromycin consumption may be a contributing factor for impaired vaccine success in decreasing resistance, as suggested in a study evaluating the effect of 7-valent pneumococcal conjugate vaccine in Portugal (*26*).

The first limitation of our study was our use of sales data on antimicrobial drugs; sales information does not guarantee that patients actually received and used the drugs. Also, we could not exclude multiple isolates obtained from the same patient in the *E. coli* database. Furthermore, we could not definitively determine that a patient had not been hospitalized shortly before sample collection. However, the importance of *E. coli* in community-acquired infections, the large number of isolates (>400,000), and the consistency of our findings suggest strong reliability of the results. Because there is no national *E. coli* monitoring system in Brazil, we obtained these data from a company with good coverage of the study area. We could not include *Staphylococcus aureus* and *Klebsiella pneumoniae* and some other microorganism species commonly found in both inpatient and outpatient settings in the analysis because it is not possible to differentiate nosocomial from community-acquired isolates.

Although this study suggests that the restriction policy on OTC antimicrobial sales influenced antimicrobial resistance, the results cannot be extrapolated to other scenarios, because different effects were observed for different countries and even for different regions within Brazil (*3*,*27*). Socioeconomic factors, prescription patterns, frequency of government inspections, and educational measures may also affect antimicrobial use. Also, although antimicrobial sales in public sector agencies were not affected by the restriction policy until 2012, we had no access to data from these agencies after this period and therefore could not evaluate how consumption of antimicrobial drugs distributed through public sector agencies influenced these findings.

In conclusion, antimicrobial drug sales from private pharmacies were associated with AMR in a community setting in a large metropolitan area in Brazil. Restricting OTC antimicrobial sales was associated with a drop in resistance to amoxicillin and trimethoprim/sulfamethoxazole but not to quinolones, macrolides, or cephalexin. Our findings suggest that strategies to reduce overdependence on antimicrobial drugs might have an effect on resistance in those drugs. However, any such strategy will likely need to be multifaceted because AMR is a complex problem.

Appendix Additional information on the impact on antimicrobial resistance of a policy restricting over-the-counter sales of antimicrobial drugs, Brazil.
