# Supplementary material for: Effect on Antimicrobial Resistance of a Policy Restricting Over-the-Counter Antimicrobial Sales in a Large Metropolitan Area, São Paulo, Brazil
Source: Emerg Infect Dis. 2022 Jan;28(1):180–7. doi: 10.3201/eid2801.201928 (PMC8714220; doi:10.3201/eid2801.201928)
Supplement: Appendix — Additional information on the impact on antimicrobial resistance of a policy restricting over-the-counter sales of antimicrobial drugs, Brazil. [file 20-1928-Techapp-s1.pdf]

Article DOI: https://doi.org/10.3201/eid2801.211928

Effect on Antimicrobial Resistance of a Policy Restricting Over-the-Counter Antimicrobial Sales in a Large Metropolitan Area, São Paulo, Brazil

Appendix

Statistical Model: Dynamic Regression by Bayesian Approach

The model considered was:

$$(Y_t - \bar{Y}_0) = \beta_t * (X_t - \bar{X}_0) + \varepsilon_t ,$$

$$\beta_{t+1} = \beta_t + W_t ,$$

Where:

t: month of information about sales and resistance, with ordered t;

Y_t: resistance to antimicrobial in instant t;

X_t: sales of antimicrobial in instant t;

$\bar{Y}_0$ e $\bar{X}_0$: mean resistance and mean sales, respectively, for all the period before December 2010;

β_t: parameter that represents the effect of antimicrobial sales on bacterial resistance in instant t, with β1 ~N(0,100).

$\varepsilon_t \sim N(0, \sigma_y^2)$ and $W_t \sim N(0, \sigma_{rw}^2)$: White-noise errors with variances σ_y^2 *and* σ_{rw}^2, respectively, considering *a priori*:

$$\sigma_y^2 \sim N_{truncated}(0, 100)$$

$$\sigma_{rw}^2 \sim N_{truncated}(0, 100)$$

Appendix Table. Antimicrobial sales in Sao Paulo metropolitan region, 2008–2016 by private pharmacies and 2008–2012 by other sources*

	Antimicrobial sales		
Year	Private pharmacies	Other channels†	Overall
2008	7.86	0.59	8.36
2009	8.15	0.59	8.43
2010	8.76	1.22	9.81
2011	7.20	1.61	8.52
2012	6.71	1.06	7.35
2013	5.89	NA	NA
2014	7.19	NA	NA
2015	7.24	NA	NA
2016	7.64	NA	NA
2017	8.56	NA	NA

*NA, not available.
†Primary care units, outpatient clinics and hospitals.

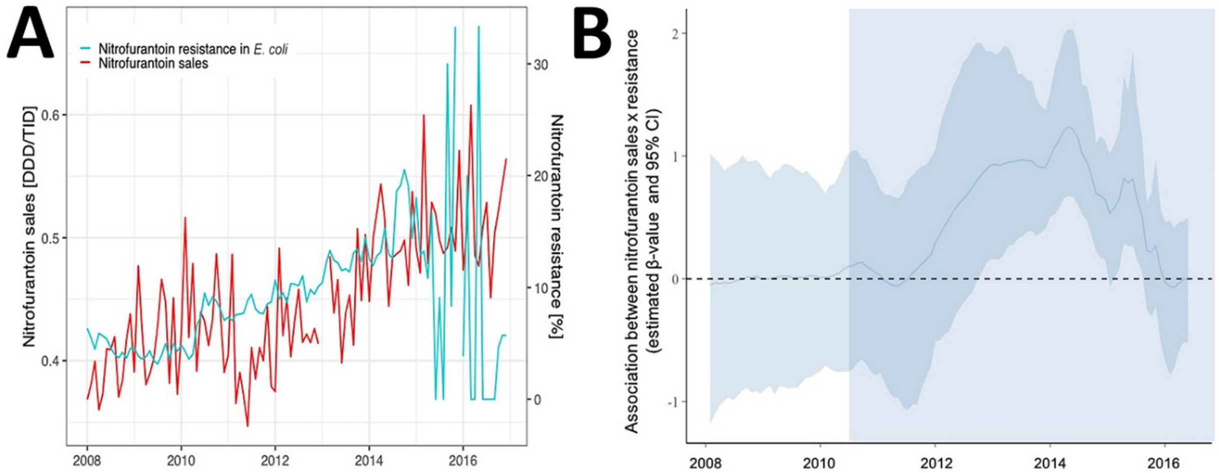

Appendix Figure 1. A) Descriptive analysis of association between nitrofurantoin sales and *E. coli* resistance for nitrofurantoin in the São Paulo metropolitan area. B) Distribution of estimated β-values obtained from dynamic regression analysis, representing the association between nitrofurantoin sales and resistance for *E. coli*. A positive value and 95% credibility interval >0 suggest a significant and direct association between sales and resistance after the implementation of restriction policy (blue shaded area). The end of the series was not fitted to the model due to lack of data. DID, defined daily dose/1,000 inhabitants/day

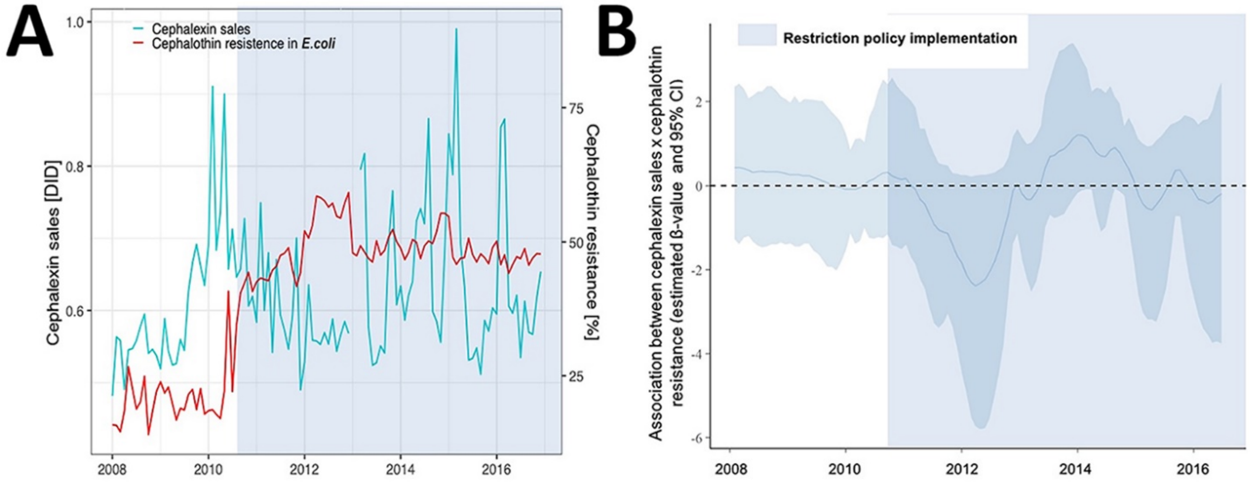

Appendix Figure 2. A) Descriptive analysis of association between cephalexin sales and *E. coli* resistance for cephalothin in the São Paulo metropolitan area. B) Distribution of estimated β-values obtained from dynamic regression analysis, representing the association between cephalexin sales and cephalothin resistance for *E. coli*. The 95% credibility interval crosses 0, suggesting there is no significant association between cephalexin sales and cephalothin resistance after implementation of the restriction policy (blue shaded area). DID, defined daily dose/1,000 inhabitants/day; 95%CI, 95% credibility interval.

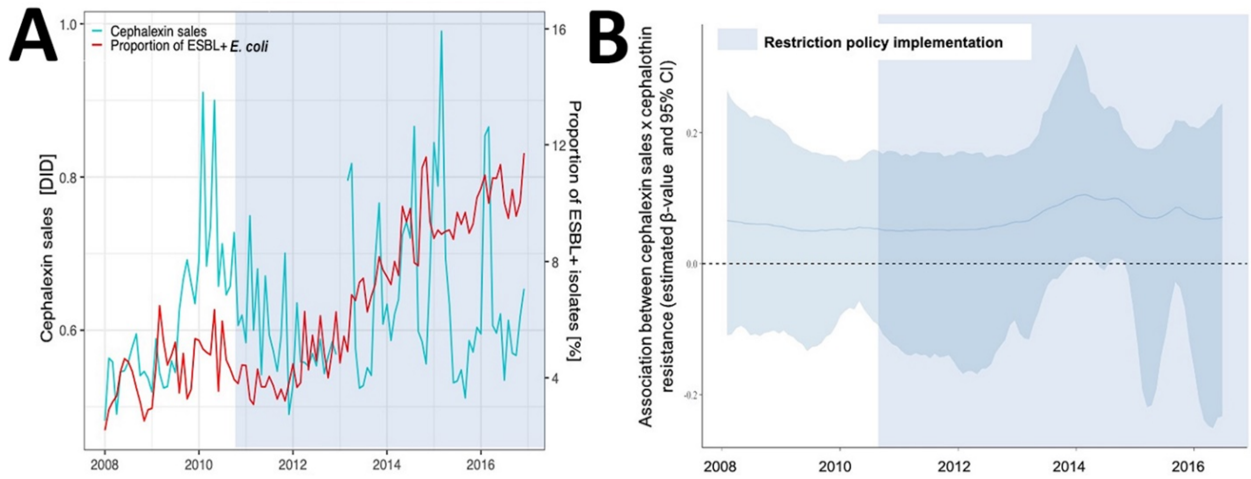

Appendix Figure 3. A) Descriptive analysis of cephalexin sales and proportion of ESBL positive *E. coli* in the São Paulo metropolitan area. B) Distribution of estimated β-values obtained from dynamic regression model, representing the association between cephalexin sales and ESBL+ *E. coli*. The 95% credibility interval crosses the zero value, suggesting there is no significant association between cephalexin sales and proportion of ESBL positive isolates after the implementation of restriction policy (blue shaded area). DID, defined daily dose/1,000 inhabitants/day; 95%CI, 95% credibility interval.
